# [^18^F]NaF PET/CT imaging of response to single fraction SABR to bone metastases from breast cancer

**DOI:** 10.3389/fnume.2023.1197397

**Published:** 2023-10-04

**Authors:** Nicholas Hardcastle, Yang Liu, Shankar Siva, Steven David

**Affiliations:** ^1^ Department of Physical Sciences, Peter MacCallum Cancer Centre, Melbourne, VIC, Australia; ^2^ Sir Peter MacCallum Department of Oncology, The University of Melbourne, Melbourne, VIC, Australia; ^3^ Centre for Medical Radiation Physics, University of Wollongong, Wollongong, NSW, Australia; ^4^ Western Health Victoria, Melbourne, VIC, Australia; ^5^ Department of Radiation Oncology, Peter MacCallum Cancer Centre, Melbourne, VIC, Australia

**Keywords:** SAbR, SBRT, breast, bone metastases, sodium fluoride, naF PET

## Abstract

Breast cancer commonly metastasises to the skeleton, and stereotactic ablative body radiation therapy (SABR) is an emerging treatment for oligometastatic disease. Accurately imaging bone metastases and their response to treatment is challenging. [^18^F]NaF-PET has a higher sensitivity and specificity than conventional bone scans for detecting breast cancer bone metastases. In this pre-defined secondary analysis of a prospective trial, we evaluated the change in [^18^F]NaF uptake after SABR. Patients with oligometastatic breast cancer received a single fraction of 20 Gy to up to three bone metastases. [^18^F]NaF-PET was acquired before and 12 months after SABR. Pre- and post-treatment [^18^F]NaF-PET images were registered to the treatment planning CT. The relative change in tumour SUV_max_ and SUV_mean_ was quantified. The intersection of each of the radiation therapy isodose contours with a non-tumour bone was created. The change in SUV_mean_ in sub-volumes of non-tumour bone receiving doses of 0–20 Gy was quantified. In total, 14 patients, with 17 bone metastases, were available for analysis. A total of 15 metastases exhibited a reduction in SUV_max_; the median reduction was 42% and the maximum reduction 82%. An increased absolute reduction in SUV_max_ was observed with higher pre-treatment SUV_max_. One patient exhibited increased SUV_max_ after treatment, which was attributed to normal peri-tumoural bone regeneration in the context of a bone metastasis. There was a median reduction of 15%–34% for non-tumour bone in each dose level.

## 1. Introduction

The skeleton is the most common site of cancer metastases from breast cancer, and a significant proportion of patients with breast cancer present with bone-only metastases (1, 2). Compared with patients with visceral metastases, those with bone-only metastases have a longer survival (3). The control of bone metastases may have a survival advantage, particularly in the oligometastatic setting. However, medical imaging techniques must be accurate and reliable for identifying bone metastases and assessing treatment responses.

There is no consensus approach in the assessment of bone metastasis, despite recommendations from MD Anderson (MDA), Union for International Cancer Control (UICC), and the World Health Organization (WHO), in addition to Positron Emission Tomography Response Criteria In Solid Tumors (PERCIST) and Response Evaluation Criteria in Solid Tumors **(**RECIST**)** guidelines (4–7). The more widely used imaging techniques for bone metastases for breast cancer are bone scintigraphy (BS) with [^99^m]Technetium-labelled diphosphonate, computed tomography (CT), 2-[^18^F]-fluoro-2-deoxy-D-glucose ([^18^F]FDG)-positron-emission tomography (PET) or single photon emission computed tomography (SPECT). The optimal imaging modality for bone metastases depends on the clinical scenario and individual tumour, with respect to lytic, blastic, and soft-tissue components and metabolic activity. For example, lytic tumours are often poorly visualised on BS, and metastases with lobular histology have reduced uptake on [^18^F]FDG PET (8, 9). CT scans only show structural components that limit visibility to metastases’ structural abnormality, requiring imaging such as BS or [^18^F]FDG PET to accurately visualise them. As such, in breast cancer bone metastases, which are often a mix of lytic and blastic types, multi-modality imaging is critical for accurate identification and visualisation.

The recent use of [^18^F] sodium fluoride ([^18^F]NaF) for the imaging of cancer in bone has been developed as a surrogate marker for the regional bone regeneration rate resulting from osteoblast, osteoclast, and osteocyte activity (10, 11). [^18^F]Fluorine was first described in 1962 as a positron-emitting bone tracer (12). Due to the development of the PET scan, it has been recently used in PET/CT. A preclinical study showed that [^18^F]NaF does not bind to protein and is absorbed by bone rapidly, facilitating the acquisition of high-contrast images of osteoblastic activity and blood flow (13). Recent studies show that compared with a bone scan using ^99m^Tc-Methyl diphosphonate (^99m^Tc MDP), [^18^F]NaF has a higher sensitivity, specificity, accuracy, and negative predictive value in detecting breast cancer metastases (14, 15).

Therefore, for patients with oligometastatic breast cancer who are suitable for locally ablative therapies such as stereotactic ablative body radiation therapy (SABR), [^18^F]NaF PET/CT can be potentially used to accurately diagnose and monitor skeletal metastases in response to treatment. This study describes [^18^F]NaF PET/CT imaging in response to single fraction SABR to bone metastases from breast cancer.

## 2. Methods

This is a pre-specified exploratory analysis of a prospective clinical trial (BOSTON, ACTRN12614000484640) (16). A total of 15 patients with oligometastatic breast cancer were enrolled in this study between October 2014 and August 2017, and received stereotactic ablative body radiation therapy to bone metastases. Patient characteristics were described by David et al. (16); briefly, the median age was 63 years, 13 patients had hormone receptor positive disease, two patients had Her-2 positive disease, and one patient had triple negative breast cancer. All patients had their primary breast cancer surgically resected, 12 patients had previous chemotherapy, 13 had previous hormone therapy, three had previous targeted therapy, and 10 had previous radiation therapy to the primary tumour. Patients with more than three metastases detected by the pre-treatment PET/CT screening were excluded from the study. It was not mandated for metastases to be biopsied due to the technical difficulty in many instances. However, all patients referred into the trial were diagnosed as oligometastatic on conventional imaging (CT and whole-body SPECT bone scan) and were reviewed at a multidisciplinary tumour stream meeting with access to all imaging.

All patients had a staging [^18^F]NaF PET/CT scan acquired before study enrolment and 12 months after treatment. Scans were acquired on either a Siemens Biograph 6 TruePoint or Biograph 16 TruePoint scanner from three centres with a 16.2 cm field of view, and reconstructed using point spread function (PSF) modelling reconstructions at two or three iterations and 21 subsets (2i21s or 3i21s) using post-reconstruction 4–8 mm Gaussian filtering and a voxel size of 3.39 mm × 3.39 mm × 3 mm (patient 1) or 4.07 mm × 4.07 mm × 5 mm (all other patients). All but four patients had both pre- and post-treatment [^18^F]NaF PET/CT acquired on the same scanner. Scanner-specific PET resolution full width at half maximum (FWHM) values are not available for this study. Based on manufacturer specifications, a Biograph Truepoint 6 typically features a 5.9 and 6.0 mm transaxial FWHM at 1 and 10 cm, respectively, and a 5.5 and 6.0 mm axial FWHM at 1 and 10 cm, respectively.

### 2.1. Radiation therapy

CT imaging for radiation therapy treatment planning was performed on a Philips Brilliance 16-slice wide-bore scanner. Images were acquired at 140 kVp with a tube current in the range of 77–391 mAs, collimation of 16 mm × 1.5 mm, rotation time of 0.44 s and reconstructed with a voxel size of 1.17 mm × 1.17 mm × 3.00 mm. The gross tumour volume (GTV) was delineated using a combination of all available imaging, including the treatment planning CT, pre-treatment [^18^F]NaF PET/CT and [^18^F]F-FDG PET/CT. A 5 mm planning target volume (PTV) margin was applied to the GTV. A single fraction of 20 Gy was prescribed to cover 99% of the PTV, aiming for a maximum dose in the GTV of 125% of the prescription dose. The calculated dose grid voxel size was 2.5 mm × 2.5 mm × 2.5 mm. Treatment was delivered with three-dimensional conformal or intensity-modulated radiation therapy. Image guidance was performed before and during treatment using planar X-rays and cone-beam CT.

### 2.2. Image response assessment

Two sets of [^18^F]NaF PET/CT scans (pre-treatment and post-treatment) and the CT scan for planning the radiation therapy with contours and dose grid were imported into MIM software (v6.6, MIM software, Cleveland, OH, USA). The CT scan for planning the radiation therapy treatment was used as the reference spatial frame of reference as it is where the tumour volume and radiation therapy doses are defined. To register both [^18^F]NaF PET/CT scans to the planning CT scan, an initial intensity-based rigid registration was applied to the whole CT dataset. Second, a 5 cm × 5 cm × 5 cm bounding box around the GTV was applied to refine the registration. Finally, manual adjustment ensured registration accuracy at the bone target. Registration accuracy was assessed manually using image fusion and line profile tools and was estimated to be accurate within one CT voxel (1.17 mm × 1.17 mm × 2 mm) for rigid bones. After all the CT images were registered, the registration was applied to the PET component of the [^18^F]NaF PET/CT scan. Therefore, the tumour and radiation therapy region were aligned on both the [^18^F]NaF PET and planning CT scans (Supplementary Figure S1). Due to the difference in orientation and rotation of target bones between scans, this registration is only accurate for locations proximal to the GTV.

#### 2.2.1. Tumour response

To measure the difference in [^18^F]NaF PET uptake before and after treatment, the maximum standardised uptake value in any voxel in the GTV contour (SUV_max_) and the mean SUV of all voxels in the GTV contour (SUV_mean_) were computed. SUV was computed normalised to body weight. The relative difference was calculated per GTV as [SUV_max−post_—SUV_max−pre_]/SUV_max−pre_. The Wilcoxon signed rank test was used to compare the distribution of GTV SUV_max_ before and after treatment. Spearman's rank correlation was computed to assess the relationship between the relative change in GTV SUV_max_ or SUV_mean_ and the pre-treatment SUV_max_ or SUV_mean_. A cutoff of *p* < 0.05 for statistical significance was chosen. The lesion response at 12 months after treatment as defined on CT and [^18^F]NaF PET scans and from clinical features was included as reported by David et al. (16) using the MD Anderson response assessment tool.

#### 2.2.2. Normal bone response

The bone was manually contoured 2 cm around the PTV contour, which was subtracted from this, resulting in proximal non-tumour bone. Radiation therapy isodose lines from 0 Gy to 24 Gy with intervals of 2 Gy were contoured. Each higher isodose contour proximal to the GTV was subtracted from its adjacent distal lower isodose contour to result in 2 Gy isodose ring contours (Supplementary Figure S1). The intersection of the isodose ring contours with the proximal non-tumour bone was derived, resulting in contours of proximal non-tumour bone receiving 2 Gy dose increments up to 24 Gy. The SUV_mean_ of non-tumour bone receiving each interval dose of 2 Gy was extracted. The change in pre- and post-treatment SUV_mean_ was calculated as [SUV_mean−post_—SUV_mean−re_]/SUV_mean−pre_.

## 3. Results

Data from 14 out of 15 patients were included in this analysis, with 17 bone metastatic lesions detected by [^18^F]NaF. The median time from pre-treatment PET to post-treatment PET was 13 months (range 11–14 months). One patient did not have the follow-up [^18^F]NaF PET due to disease progression before this assessment and was not included in the analysis. In one patient (patient 5, right humerus head), the humerus and the scapula were registered separately for non-tumour bone analysis due to the different rotation of the humerus head compared with the scapula, which is within the 2 cm range from the PTV.

### 3.1. Tumour response

**Figure 1:**
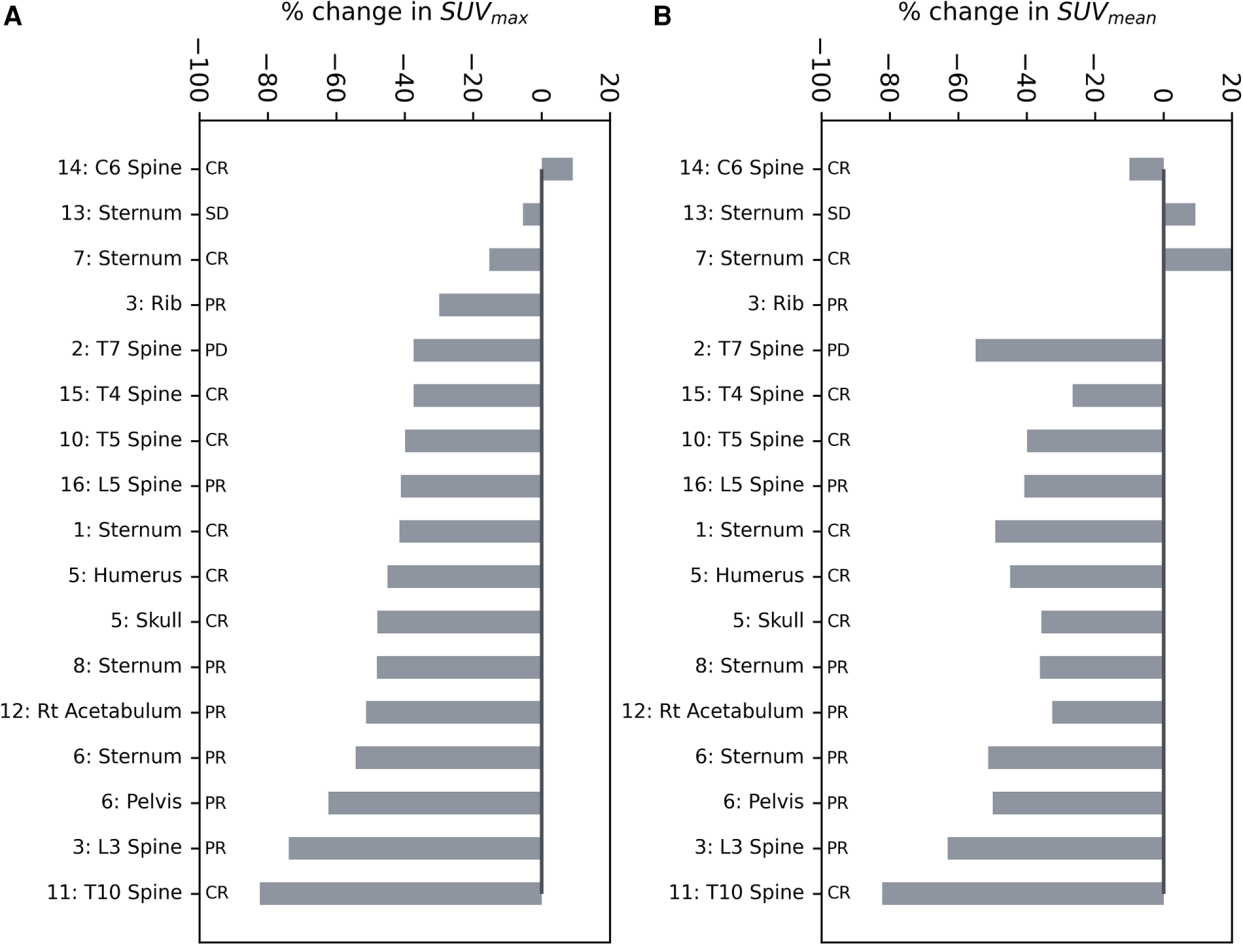
Waterfall plots of the relative change in (**A**) SUV_max_ and (**B**) SUV_mean_ of the GTV after SABR. The outcome of the lesion based on all available clinical and imaging data is provided. CR, complete response; SD, stable disease; PR, partial response; PD, progressive disease.

The relative change of SUV_max_ and SUV_mean_ [^18^F]NaF uptake at 12 months after SABR in the GTV was plotted as a waterfall plot (Figure 1); 15 out of 17 bone lesions show a reduction in SUV_max_, with the maximal reduction up to 82%, and 14 out of 17 bone lesions had a reduction in SUV_mean_, with the maximum reduction of 82%. The median GTV SUV_max_ was 25 (range 7–108) before treatment and 15 (range 3.9–60) after treatment (*p* ≤ 0.005), and the median GTV SUV_mean_ was 10 (range 4–40) before treatment and 6.2 (range 3–18) after treatment (*p* = 0.002). The absolute reduction in SUV_max_ and SUV_mean_ increased with increasing pre-treatment SUV_max_ and SUV_mean_, respectively (*p* << 0.005 for both SUV_max_ and SUV_mean_) (Supplementary Figure S2). The median GTV mean HU was 275 (range 48–795) before treatment and 277 (range −7 to 716) after treatment (*p* = 0.40) (Supplementary Table S1).

**Figure 2:**
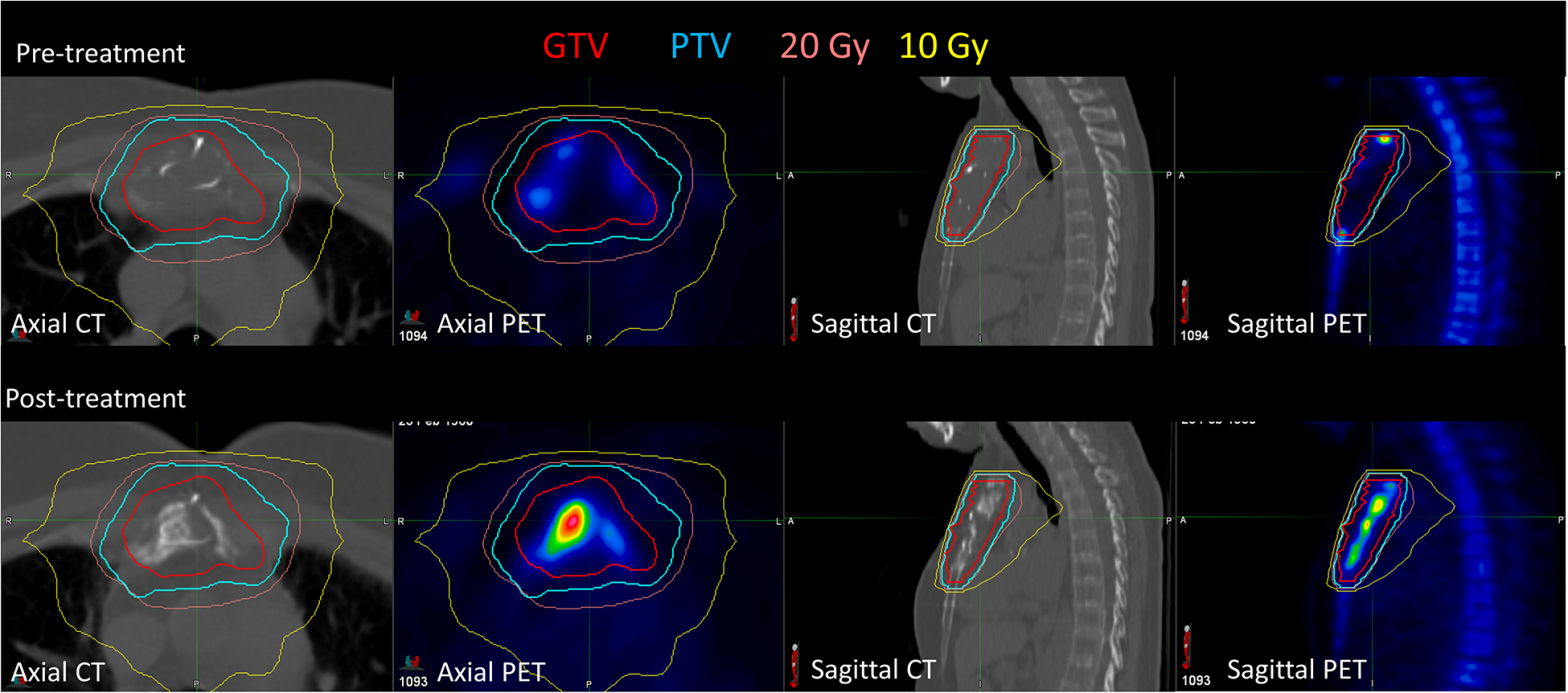
Pre-treatment and post-treatment CT and [^18^F]NaF PET for patient 7, who had a sternal metastasis treated. After treatment, [^18^F]NaF SUV_max_ decreased from 36.4 to 30.8, and SUV_mean_ increased from 3.5 to 3.9, corresponding to bone regeneration.

**Figure 3:**
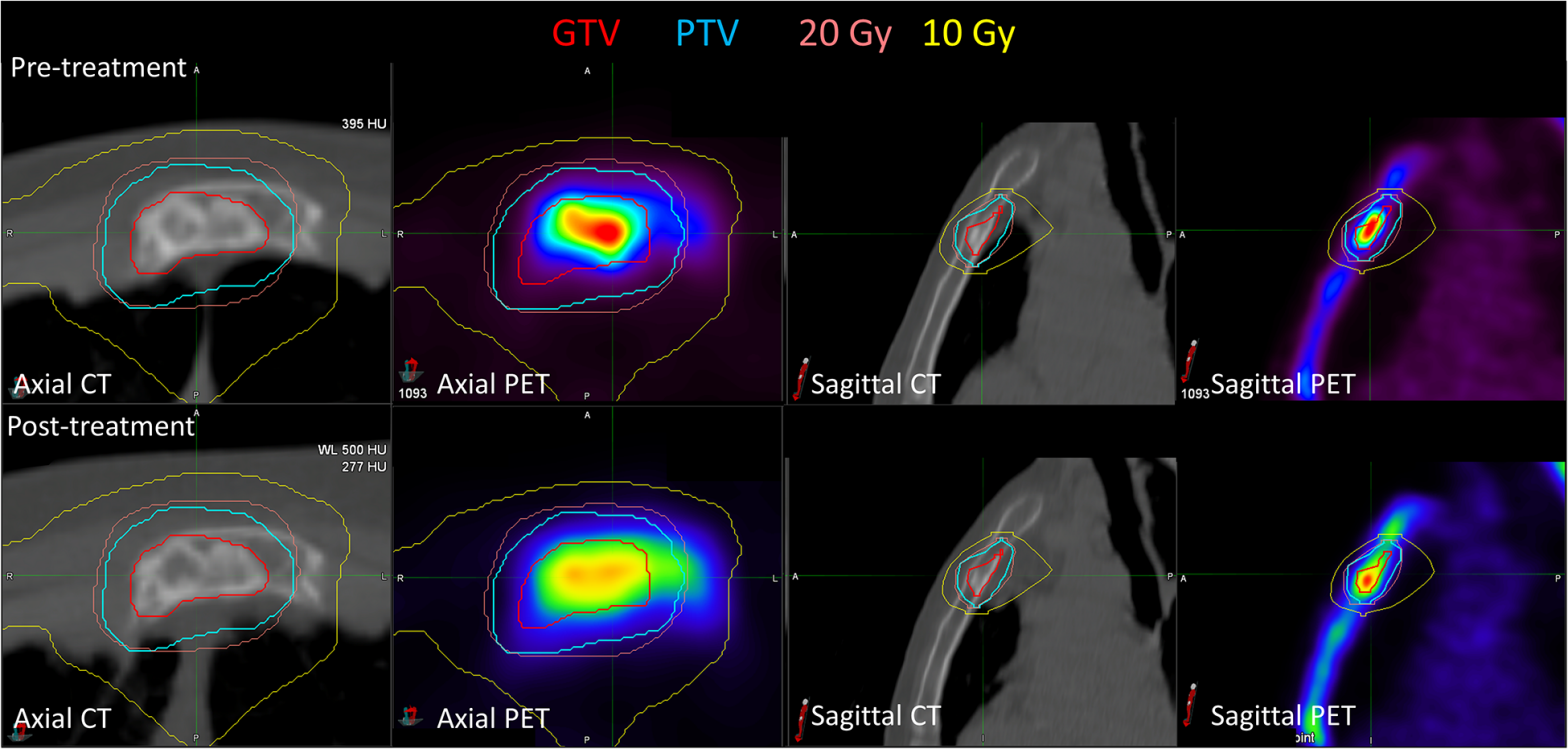
Pre-treatment and post-treatment CT and [^18^F]NaF PET for patient 13, who had a sternal metastasis treated. Both SUV_max_ (decreased from 18.0 to 17.0) and SUV_mean_ (increased from 8.7 to 9.5) were relatively stable post-treatment. There were minimal observable CT changes consistent with minimal bone regeneration.

Figure 2 shows the pre- and post-treatment imaging for patient 7, who had a predominantly lytic lesion of the sternum with low initial [^18^F]NaF uptake, with the exception of the superior aspect of the GTV. At 12 months after treatment, the SUV_max_ decreased from 36.4 to 30.8, but a significant increase in [^18^F]NaF SUV_mean_ (3.5 increasing to 5.9) was observed, corresponding to bone regeneration and complete response; the mean Hounsfield Unit increased from 58 to 162 in the GTV, indicating increased bone component within GTV. Conversely, patient 13 also had a sternal metastasis treated; there was minimal change in [^18^F]NaF uptake; SUV_max_ decreased minimally from 18 to 17, and SUV_mean_ slightly increased from 8.7 to 9.5, indicating stable disease (Figure 3).

**Figure 4:**
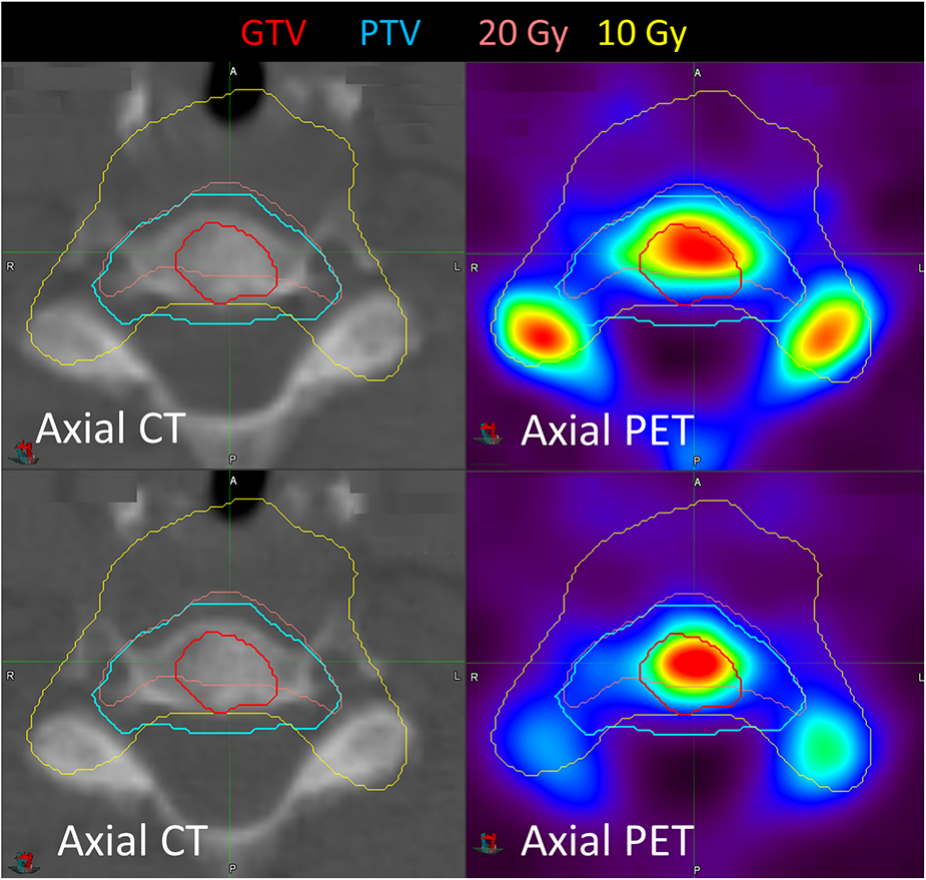
Pre- and post-treatment imaging for patient 14 who had a C6 metastasis treated. SUV_max_ increased after treatment from 11.0 to 12.0, but SUV_mean_ decreased from 7.0 to 6.3.

Three patients had two metastases treated. Patient 3 had an L3 vertebra and a rib metastasis treated. The pre-treatment SUV_max_ was significantly higher for the L3 vertebra (77.6) than the rib (11.0), indicating substantial heterogeneity in uptake for this patient. Patient 6 had pelvic and sternal metastases treated, with a pre-treatment SUV_max_ of 25.0 and 18.2, respectively, and patient 5 had humeral and skull metastases treated, with a SUV_max_ of 7.1 and 16.0, respectively. Figure 4 shows the imaging for patient 14, who had a C6 metastasis treated. The 20 Gy prescription isodose line did not cover the full GTV due to the proximity of the spinal cord; a relatively stable SUV_max_ (11.0 increasing to 12.0) and SUV_mean_ (7.0 decreasing to 6.3) were observed. Despite this, there has been no progression in this treated vertebra.

### 3.2. Non-tumour bone response

The change in SUV_mean_ was calculated for non-tumour bone adjacent to each of the 17 treated metastases. Supplementary Figure S3 shows the change in SUV_mean_ in non-tumour bone as a function of dose. The median SUV_mean_ across all patients decreased in each isodose contour. There was, however, no correlation between the change in SUV_mean_ as a function of the delivered dose to the non-tumour bone.

## 4. Discussion

The imaging of bone metastases for treatment response is challenging due to a wide range of available imaging modalities, each with limitations and a lack of consensus guidelines for the assessment of response. MDA, UICC, and the WHO have developed bone metastasis response criteria, and RECIST only considers bone disease measurable if it contains a soft-tissue component >10 mm (17). Many imaging modalities are used, including X-ray, scintigraphy, magnetic resonance imaging (MRI) and PET. In this context, [^18^F]NaF PET has improved sensitivity (94.2%–100%) and specificity (46.3%–97%) over conventional bone SPECT (14, 15).

The present study evaluates the tumour-specific response of breast cancer bone metastases to a single fraction of high-dose radiation using [^18^F]NaF PET/CT. Overall, [^18^F]NaF uptake was significantly reduced after single fraction SABR. The decrease of [^18^F]NaF, however, was dependent on the initial uptake, with those metastases with the highest uptake having the largest visible response. Similar heterogeneity in [^18^F]NaF response has been demonstrated in bone metastases from breast cancer treated with systemic therapy (18–20). Although metastases deemed to be progressing based on conventional imaging typically exhibited increased [^18^F]NaF uptake, there was heterogeneity in response. This includes non-progressing lesions exhibiting an increase in [^18^F]NaF uptake attributed to treatment-induced flare, resulting in limitations in the ability of [^18^F]NaF for response assessment. We observed only two patients with increased [^18^F]NaF uptake after treatment, which is potentially lower than that observed in the previously mentioned systemic therapy response studies. A potential reason may be related to the lesion type; all metastases, with the exception of the sternal metastasis in patient 7, were classified as mixed lytic and sclerotic components. Patient 7, however, had a very large lytic volume. Second, the use of [^18^F]NaF for response assessment after systemic therapy was typically performed at 8–12 weeks after the commencement of treatment, compared with 12 months in our study. This later imaging time point may be too late to observe post-treatment bone regeneration in all but those in whom substantial bone regeneration after treatment occurs. The heterogeneity in response due to non-tumour factors, such as bone regeneration, indicates the need for multi-modality imaging for response assessment in bone metastases with experience from both nuclear medicine physicians and radiologists essential in the interpretation of metastatic disease treated with SABR.

Our previous study evaluated [^18^F]NaF for prostate cancer bone metastases (21). Between the two studies, the uptake was very similar; the median SUV_max_ and SUV_mean_ were 26 ( range 4–112) and 13 (range 4–41), respectively, for prostate cancer metastases compared with 25 (range 7–108) and 10 (range 4–40) for breast cancer metastases. The mean reduction in SUV_max_ for prostate cancer metastases was 17.7; in the present study, it was 18.7 for breast cancer metastases.

[^18^F]FDG PET is now emerging as a more widely used staging tool in metastatic breast cancer as it assesses both bone and visceral disease simultaneously and is now funded in many countries for diagnostic staging and response assessment (22, 23). The addition of [^18^F]NaF PET scanning may, however, improve the detection of bone metastases (24), but there is limited evidence exploring its efficacy in response assessment. The role of SABR in oligometastatic breast cancer is currently an area of ongoing research, with recent reports containing mixed and conflicting results regarding its efficacy (25, 26). Further research in breast cancer specific trials is ongoing (27–31). Future research in this area is likely to be biomarker driven both for the selection of patients and monitoring the response to treatment. This study demonstrates the usefulness of [^18^F]NaF PET in response assessment, and further research is required to define its role as a potential biomarker in both accurately identifying oligometastatic patients and their response to SABR.

The present study has some limitations. Although there were only three scanners used in this study, two of them were in community private practice and none of the scanners were harmonised. We do not have values for the resolution at FWHM for the specific scanners used in this study. This may potentially limit the interpretation of the absolute SUVs due to the variability introduced in absolute counts. However, of the 14 included patients, 10 were scanned on the same scanner for pre- and post-treatment imaging, limiting the impact of variation in SUV between imaging sessions to levels expected in a test–retest scenario. The repeatability of [^18^F]NaF PET in a test–retest scenario has previously been quantified in harmonised scanners, demonstrating a coefficient of variability in SUV_max_ and SUV_mean_ of 14.1% and 6.6%, respectively (32). It should also be noted that an evaluation of regional bone plasma clearance (*K_i_*) was not performed in this study, which, although is more challenging to measure, may provide different results from those achieved in this work based on SUV (11, 33). A further potential limitation arises from the spatial registration between scans. We elected to use the CT scan planning the radiation therapy treatment as the spatial frame of reference; however, resampling the PET data to match the planning CT may have a minor impact on PET uptake values. Lastly, as stated above, [^18^F]FDG PET would ideally be acquired for this patient cohort, but unfortunately this trial pre-dated the routine reimbursement of this imaging in this patient cohort.

## 5. Conclusion

In the context of breast bone oligometastases treated by a high-dose single fraction SABR, [^18^F]NaF uptake was reduced in 15 out of 17 bone lesions. An increased [^18^F]NaF uptake was observed for one lytic lesion, which was correlated with bone regeneration. There was reduced uptake in adjacent non-tumour bone receiving high doses. Further research is required to explore the use of [^18^F]NaF PET in conjunction with other imaging modalities to more accurately assess treatment response.

## Data Availability

The raw data supporting the conclusions of this article will be made available by the authors, without undue reservation.

## Supplementary material

Supplementary Figure S1 Workflow to register pre- and post-treatment [18F]NaF PET-CT images to the radiation therapy treatment planning CT. The CT components of the PET/CT scans were rigidly registered to the treatment planning CT. The registration was applied to the PET data to obtain the PET data on the treatment planning CT, on which the tumour structure and the planned radiation dose was defined.

Supplementary Figure S2 Absolute change in SUV_max_ and SUV_mean_ in each of the GTVs after treatment, as a function of their pre-treatment values.

Supplementary Figure S3 Box plots of the relative change in SUV_mean_ in non-GTV bone around each of the tumours as a function of the planned radiation therapy isodose to that bone.

Supplementary Table 1 Change in mean CT number in the GTV from pre- to post-treatment.
