# Supplementary figures and images for: [18F]NaF PET/CT imaging of response to single fraction SABR to bone metastases from breast cancer
Source: Front Nucl Med. 2023 Oct 4;3:1197397. doi: 10.3389/fnume.2023.1197397 (PMC11460292; doi:10.3389/fnume.2023.1197397)

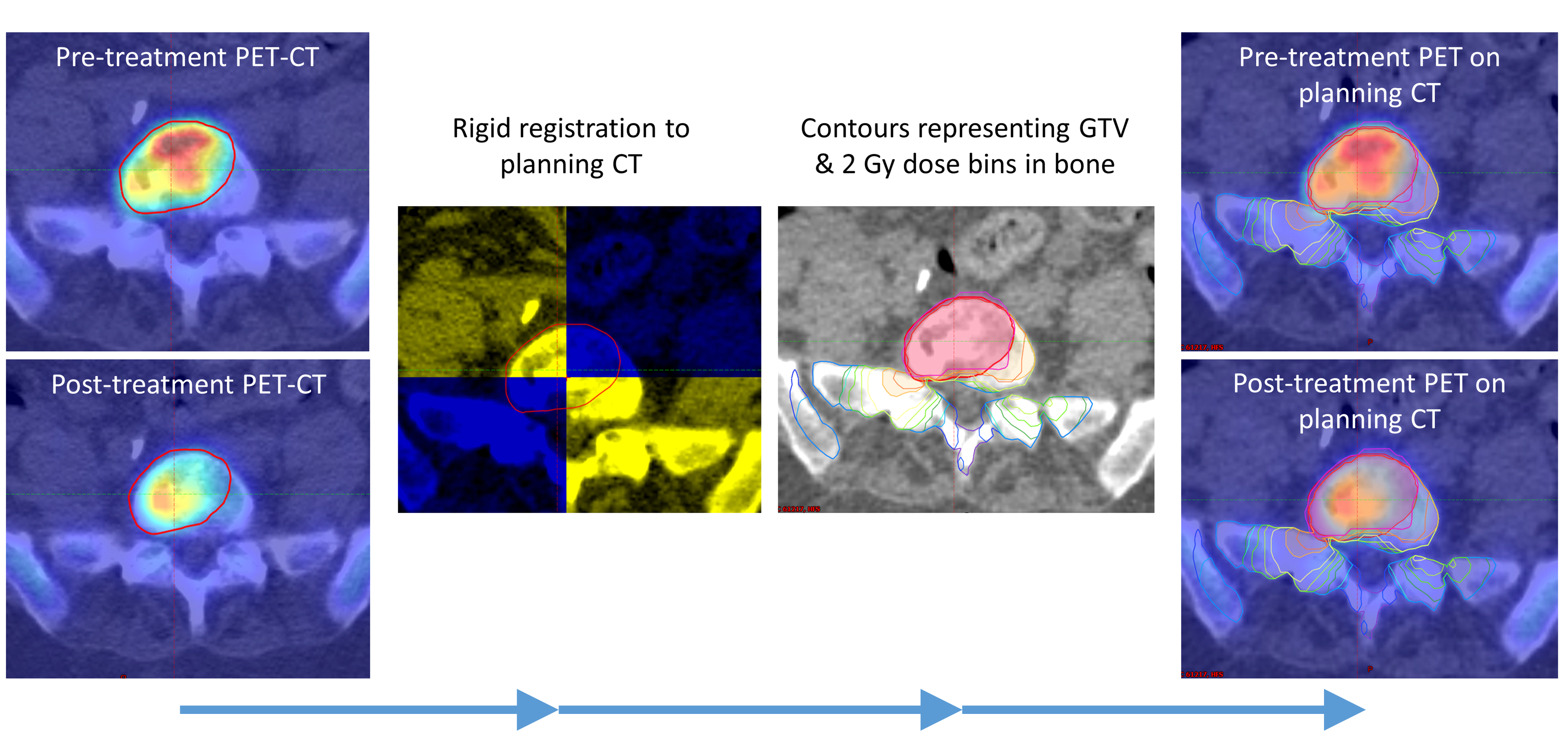

Supplement: Supplementary Figure S1 — Workflow to register pre- and post-treatment [18F]NaF PET-CT images to the radiation therapy treatment planning CT. The CT components of the PET/CT scans were rigidly registered to the treatment planning CT. The registration was applied to the PET data to obtain the PET data on the treatment planning CT, on which the tumour structure and the planned radiation dose was defined. [file Image1.tif]

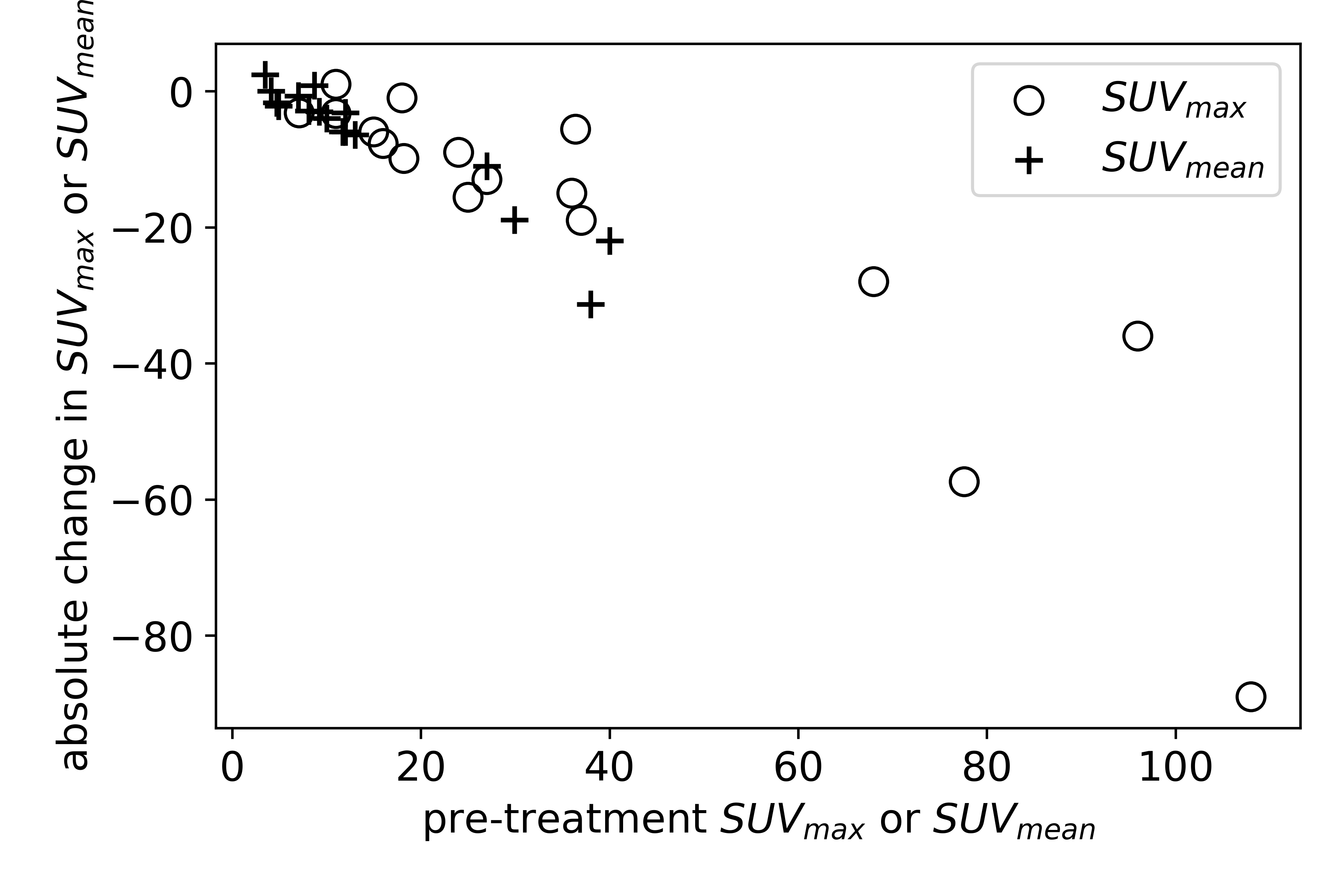

Supplement: Supplementary Figure S2 — Absolute change in SUVmax and SUVmean in each of the GTVs after treatment, as a function of their pre-treatment values. [file Image2.tif]

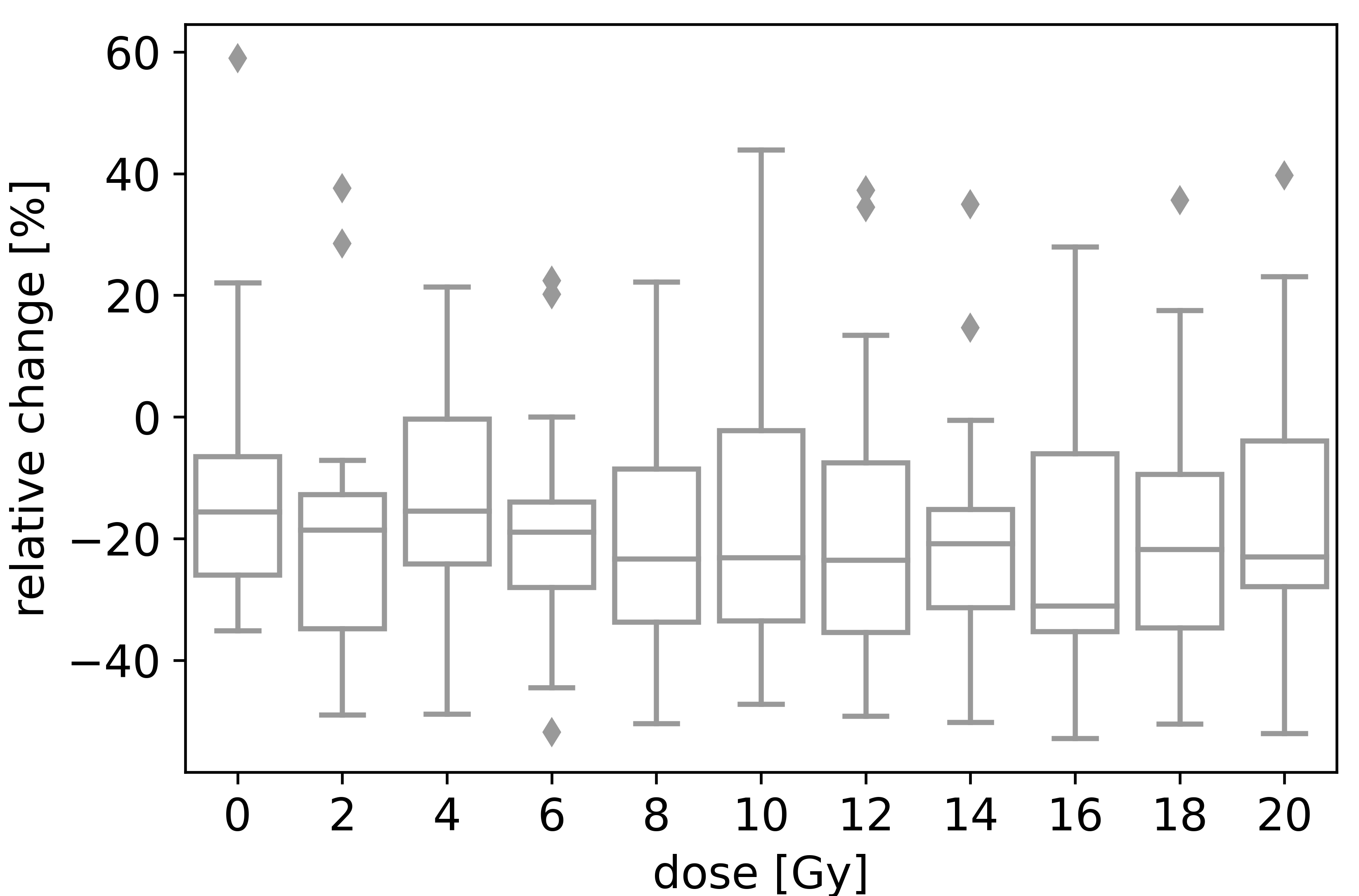

Supplement: Supplementary Figure S3 — Box plots of the relative change in SUVmean in non-GTV bone around each of the tumours as a function of the planned radiation therapy isodose to that bone. [file Image3.tif]
