# Supplementary material for: [18F]NaF PET/CT imaging of response to single fraction SABR to bone metastases from breast cancer
Source: Front Nucl Med. 2023 Oct 4;3:1197397. doi: 10.3389/fnume.2023.1197397 (PMC11460292; doi:10.3389/fnume.2023.1197397)
Supplement: Supplementary Table 1 — Change in mean CT number in the GTV from pre- to post-treatment. [file Table1.docx]

| Patient: Tumour | Mean CT number in the GTV | | Change (post - pre) |
| --- | --- | --- | --- |
|  | Pre-treatment | Post-treatment |  |
| 1: Sternum | 457 | 602 | 145 |
| 10: T5 Spine | 306 | 277 | -29 |
| 11: T10 Spine | 256 | 397 | 141 |
| 12: Rt Acetabulum | 494 | 491 | -3 |
| 13: Sternum | 509 | 480 | -29 |
| 14: C6 Spine | 552 | 567 | 15 |
| 15: T4 Spine | 275 | 261 | -14 |
| 16: L5 Spine | 222 | 228 | 6 |
| 2: T7 Spine | 601 | 716 | 115 |
| 3: L3 Spine | 254 | 357 | 103 |
| 3: Rib | 173 | 223 | 50 |
| 5: Humerus | 48 | -7 | -55 |
| 5: Skull | 795 | 596 | -199 |
| 6: Pelvis | 313 | 276 | -37 |
| 6: Sternum | 146 | 108 | -38 |
| 7: Sternum | 58 | 162 | 104 |
| 8: Sternum | 158 | 196 | 38 |
